# A Strategy for Cultivation of Retargeted Oncolytic Herpes Simplex Viruses in Non-cancer Cells

**DOI:** 10.1128/JVI.00067-17

**Published:** 2017-04-28

**Authors:** Valerio Leoni, Valentina Gatta, Costanza Casiraghi, Alfredo Nicosia, Biljana Petrovic, Gabriella Campadelli-Fiume

**Affiliations:** a Department of Experimental, Diagnostic and Specialty Medicine, University of Bologna, Bologna, Italy; b Nouscom SRL, Rome, Italy; University of California, Irvine

**Keywords:** HER2, HSV, retargeting, Vero, oncolytic viruses

## Abstract

The oncolytic herpes simplex virus (HSV) that has been approved for clinical practice and those HSVs in clinical trials are attenuated viruses, often with the neurovirulence gene γ_1_34.5 and additional genes deleted. One strategy to engineer nonattenuated oncolytic HSVs consists of retargeting the viral tropism to a cancer-specific receptor of choice, exemplified by HER2 (human epidermal growth factor receptor 2), which is present in breast, ovary, and other cancers, and in detargeting from the natural receptors. Because the HER2-retargeted HSVs strictly depend on this receptor for infection, the viruses employed in preclinical studies were cultivated in HER2-positive cancer cells. The production of clinical-grade viruses destined for humans should avoid the use of cancer cells. Here, we engineered the R-213 recombinant, by insertion of a 20-amino-acid (aa) short peptide (named GCN4) in the gH of R-LM113; this recombinant was retargeted to HER2 through insertion in gD of a single-chain antibody (scFv) to HER2. Next, we generated a Vero cell line expressing an artificial receptor (GCN4R) whose N terminus consists of an scFv to GCN4 and therefore is capable of interacting with GCN4 present in gH of R-213. R-213 replicated as well as R-LM113 in SK-OV-3 cells, implying that addition of the GCN4 peptide was not detrimental to gH. R-213 grew to relatively high titers in Vero-GCN4R cells, efficiently spread from cell to cell, and killed both Vero-GCN4R and SK-OV-3 cells, as expected for an oncolytic virus. Altogether, Vero-GCN4R cells represent an efficient system for cultivation of retargeted oncolytic HSVs in non-cancer cells.

**IMPORTANCE** There is growing interest in viruses as oncolytic agents, which can be administered in combination with immunotherapeutic compounds, including immune checkpoint inhibitors. The oncolytic HSV approved for clinical practice and those in clinical trials are attenuated viruses. An alternative to attenuation is a cancer specificity achieved by tropism retargeting to selected cancer receptors. However, the retargeted oncolytic HSVs strictly depend on cancer receptors for infection. Here, we devised a strategy for *in vitro* cultivation of retargeted HSVs in non-cancer cells. The strategy envisions a double-retargeting approach: one retargeting is via gD to the cancer receptor, and the second retargeting is via gH to an artificial receptor expressed in Vero cells. The double-retargeted HSV uses alternatively the two receptors to infect cancer cells or producer cells. A universal non-cancer cell line for growth of clinical-grade retargeted HSVs represents a step forward in the translational phase.

## INTRODUCTION

There is an intense interest in viruses as therapeutic agents against cancer; these are termed oncolytic viruses, and there is an even greater interest in oncolytic viruses that can be administered in combination with immunotherapeutic compounds or that encode immunotherapeutic molecules (1–4). The latter include antibodies that block immune checkpoint molecules, like CTLA-4, PD-1, and PD-L1, which suppress T cell functions, as well as some cytokines and chemokines which have demonstrated immunotherapeutic activity against a variety of malignancies (5–7). Of all the oncolytic viruses that have been, or are being, tested in clinical trials, the herpes simplex virus (HSV) derivative, initially named ONCO-VEX_GM-CSF_ or T-VEC (8), went through a phase 3 clinical trial (9) and was subsequently approved by both the Food and Drug Administration and the European Medicines Agency (10). It has entered clinical practice against metastatic melanoma and is being tested against a variety of tumors, including metastatic liver, triple-negative, and head and neck cancers, and also in combination with immune checkpoint inhibitors (https://www.clinicaltrials.gov/ct2/show/NCT02509507?term=imlygic&rank=10; https://www.clinicaltrials.gov/ct2/show/NCT02779855?term=imlygic&rank=2; https://www.clinicaltrials.gov/ct2/show/NCT02626000?term=imlygic&rank=13). ONCO-VEX_GM-CSF_ represents the prototypic oncolytic HSV and is characterized by a combination of attenuation and cancer specificity, and hence by a high safety profile. The attenuation was achieved through the deletion of the virulence genes γ_1_34.5. The further deletion of the α47 gene, designed to enable a better presentation of antigenic peptides by major histocompatibility class II peptides, placed the US11 gene under the control of an immediate-early promoter (11). This conferred a higher virus replication rate than that of viruses carrying deletion of the γ_1_34.5 genes and an intact α47 gene (12). The attenuation may limit the ability of ONCO-VEX_GM-CSF_ to replicate in some cancer genotypes (13), and even more so since cancer cells are highly heterogeneous. Importantly, ONCO-VEX_GM-CSF_ encodes granulocyte-macrophage colony-stimulating factor (GM-CSF), which boosts activation of monocytes, antigen-presenting cells, and macrophages. ONCO-VEX_GM-CSF_ and all of the Δγ_1_34.5 attenuated oncolytic HSVs gain their cancer specificity, and hence their safety profile, from the fact that most cancer cells are defective in mounting innate immune responses against the viruses with these deletions.

An alternative strategy to achieve cancer specificity consists of retargeting the virus tropism to cancer-specific receptors, coupled with detargeting from the natural receptors (14–19). These viruses do not carry any other modification than those that enable the tropism retargeting. Hence, they maintain the full replication and lytic capacities of wild type (wt) HSV. The cancer receptor we selected as the target is the human epidermal growth factor receptor 2 (HER2), a member of the epidermal growth factor receptor (EGFR) family, which is overexpressed in about 25 to 30% of breast and ovarian cancers as well as in a variety of other tumors, including stomach cancer, lung cancer, and glioblastoma (20). The full retargeting requires two steps: first, the deletion or insertional inactivation of the appropriate portions of the viral envelope glycoprotein gD, the major determinant of HSV tropism which is critical for the virus interaction with its two major receptors, nectin-1 and herpesvirus entry mediator (HVEM) (15, 17, 18); second, the replacement of the deleted sequences with a ligand specific for the selected cancer receptor—in our case, a single-chain antibody (scFv) to HER2. The deleted portions of gD were amino acids (aa) 6 to 38 in the recombinant HSV construct named R-LM113 and the entire gD core (aa 61 to 218) in the recombinant HSV construct named R-LM249 (14, 15). In preclinical studies, R-LM113 and R-LM249 exerted antitumor activities against HER2-positive breast and ovarian cancers and against glioblastoma (14, 16, 21–23). Recently, we showed that not only gD but also gH can be a retargeting tool. Thus, insertion of the scFv to HER2 in gH, coupled with the deletion of aa 6 to 38 in gD, led to a fully retargeted oncolytic HSV (24).

The HER2-retargeted virus stocks employed in the preclinical studies consisted of viruses grown in the HER2-positive SK-OV-3 ovarian tumor cell line. Inasmuch as cancer cells may not be approvable for the growth of clinical-grade oncolytic HSVs (o-HSVs) and the retargeted HSVs require the HER2 oncogene to infect cells, we reasoned that even a non-cancer cell line, transgenically expressing HER2, might not be considered approvable by the regulatory agencies.

Here, we describe a strategy to grow an oncolytic HSV, retargeted to a cancer receptor and strictly dependent on it, in a non-cancer cell line. The strategy rests on a double-retargeted HSV and the construction of an *ad hoc* cell line. Briefly, the double retargeting takes advantage of the fact that not only gD but also gH are suitable tools for retargeting (24). We engineered the 20-aa-long GCN4 peptide in gH of R-LM113 which, in turn, carried the scFv to HER2 in gD. The resulting recombinant was named R-213. The *ad hoc* cell line was derived from Vero cells by expression of an artificial receptor, named Vero-GCN4R, capable of interacting with the GCN4 peptide. We report that R-213 grows at high titers in both the Vero-GCN4R cell line and in the HER2-positive cancer cell lines.

## RESULTS

### Engineering of the GCN4 peptide in the gH of the HER2-retargeted R-LM113.

**FIG 1:**
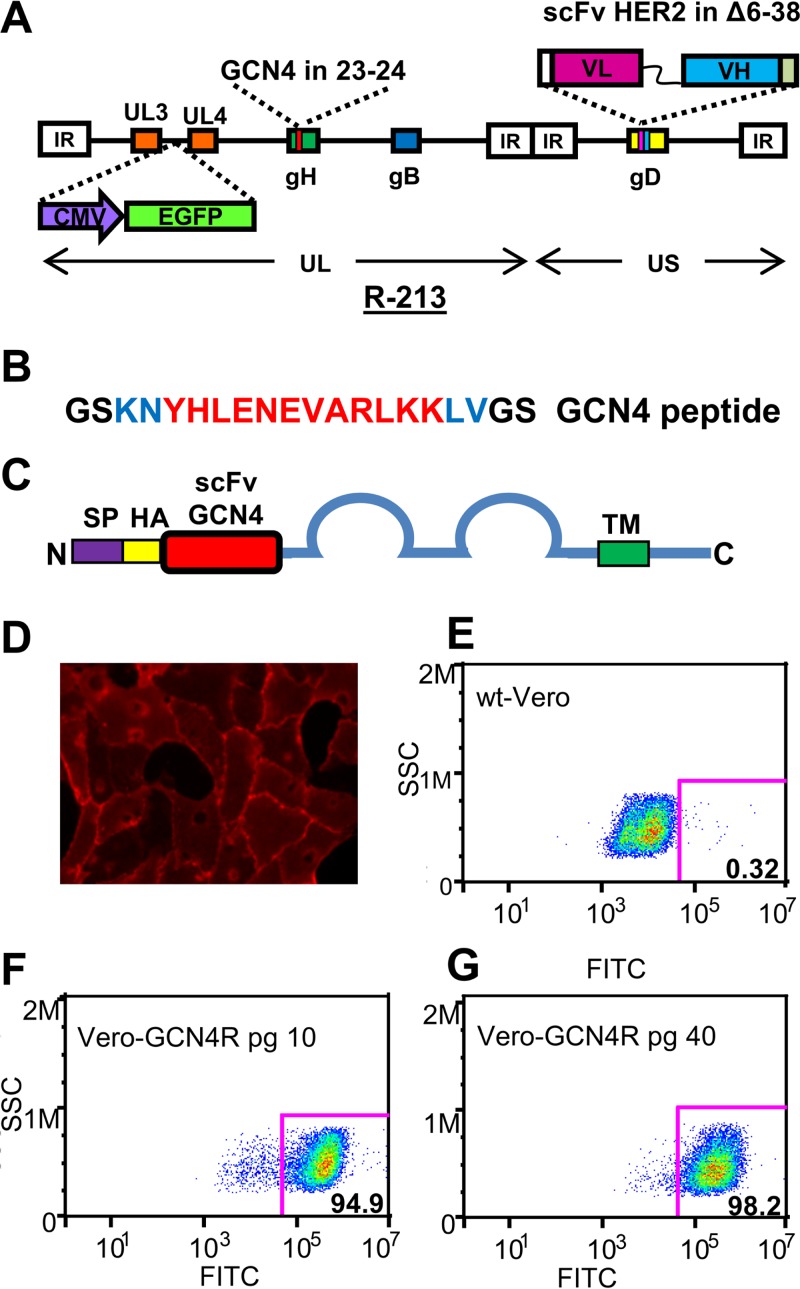
Genome organization of R-213 and generation and properties of the Vero-GCN4R cell line. (A) Genome organization of R-213. The sequence arrangement of the R-213 genome shows the inverted repeat sequences as rectangular boxes. The scFv-HER2 sequence (V_L_-linker-V_H_), bracketed by upstream and downstream linkers, is inserted in place of the deletion of aa 6 to 38 in gD. LOX-P-bracketed pBelo-BAC and EGFP sequences are inserted in the UL3-UL4 intergenic region. The sequence encoding the GCN4 peptide is engineered in gH, between aa 23 and 24. (B) Amino acid sequence of the GCN4 peptide. The epitope residues are in red, two residues flanking the epitope in wt GCN4 transcription factor are in blue, and upstream and downstream Gly-Ser linkers are in black. (C) Schematic drawing of the GCN4R chimeric receptor. The receptor is formed by an N-terminal signal peptide (purple box), an HA tag sequence (yellow box) from pDisplay (Thermo Fisher Scientific), the scFv to GCN4 (red box) bracketed by two short linkers, GA and GSGA, optimized for human codon usage, the human nectin-1 residues from Met_143_ to Val_517_, which includes the nectin-1 extracellular domains 2 and 3, the transmembrane (TM) segment (green box), and the cytoplasmic tail. (D to G) Expression of GCN4R and stability of the Vero-GCN4R cell line. The expression of the scFv GCN4-nectin receptor was analyzed by indirect immunofluorescence (D) or by fluorescence-activated cell sorting (E to G) by using a MAb to the HA tag. Diagrams show the percentages of positive cells in cultures of wt-Vero (E) or Vero-GCN4R cells at passages 10 (F) and 40 (G). SSC, side scatter; FITC, fluorescein isothiocyanate. The illustration in panel D was adjusted as follows: increase in brightness of 10%, increase in contrast of 20%.

We engineered the 20-aa-long peptide named GCN4 in the gH of R-LM113 (Fig. 1A). This peptide (25) is part of the Saccharomyces cerevisiae transcription factor GCN4, a 282-aa-long protein that belongs to the leucine zipper family and is involved in yeast amino acid synthesis. The sequence and properties of this peptide (GenBank accession number NC_001137.3) are described in reference 26. Our selection of the GCN4 peptide was based on three properties, namely, (i) this peptide is not expressed in mammalian or human cells, (ii) the sequence of the reacting scFv was available in the Protein Data Bank (PDB ID 1P4B), and (iii) the scFv bound the GCN4 peptide with very high affinity (20 pM). The GCN4 peptide has the sequence GSKNYHLENEVARLKKLVGS. The core amino acids (YHLENEVARLKK) (Fig. 1B, residues shown in red) constitute the epitope recognized by scFv C11L34-H6 (25). The core amino acids are bracketed by two flanking residues present in the original GCN4 transcription factor (Fig. 1B, residues in blue). We included upstream and downstream glycine-serine-rich (GS) linkers (Fig. 1B, residues in black) to confer flexibility. The parental R-LM113 carries the deletion of aa 6 to 38 in gD, which has been replaced by an scFv to HER2 (15). These modifications in gD detarget HSV tropism from the natural gD receptors and retarget the virus tropism to HER2. The schematic drawing of the resulting R-213 recombinant is shown in Fig. 1A.

To engineer R-213, the oligonucleotide encoding the GCN4 peptide and the GS linkers were engineered in the gH of the bacterial artificial chromosome (BAC) LM113, between aa 23 and 24 (Fig. 1A). The recombinant BAC 213 DNA was transfected into the Vero-GCN4R cell line. The reconstituted R-213 recombinant virus was grown in the same cells.

### Generation of the Vero-GCN4R cell line.

To generate the Vero-GCN4R cell line, the scFv derived from a monoclonal antibody that binds the GCN4 peptide with 20 pM affinity (25) was engineered as the N-terminal domain of an artificial receptor, herein named GCN4R. Upstream of the scFv, we included the murine Ig κ-chain leader sequence, which directs the protein to the secretory pathway (pDisplay; Invitrogen), and a hemagglutinin (HA) tag. Downstream of the scFv, the artificial receptor consisted of domains II and III, the transmembrane, and cytoplasmic tail of nectin-1 (Fig. 1C, schematic drawing). The resulting receptor was cloned in pcDNA3.1/Hygro(+) vector, under control of a cytomegalovirus (CMV) promoter, thus generating the scFv GCN4-nectin plasmid.

wt-Vero cells were transfected with the scFv GCN4-nectin plasmid, selected for resistance to 400 μg/ml hygromycin, enriched by use of microbeads, and then cloned by limiting dilution. The cell surface expression of the artificial GCN4R was detected in an indirect immunofluorescence assay (IFA) with the HA tag (Fig. 1D). To evaluate the genetic stability of the Vero-GCN4R cell line, the cells were cultured for 40 consecutive passages. Figure 1E to G shows that the percentages of HA-positive cells at passage 10 and at passage 40 were 94.9 and 98.2, respectively. This indicated that the Vero-GCN4R cell line was highly stable with respect to the cell surface expression of GCN4R.

### Double tropism of R-213 for Vero-GCN4R and HER2-positive cells.

**FIG 2:**
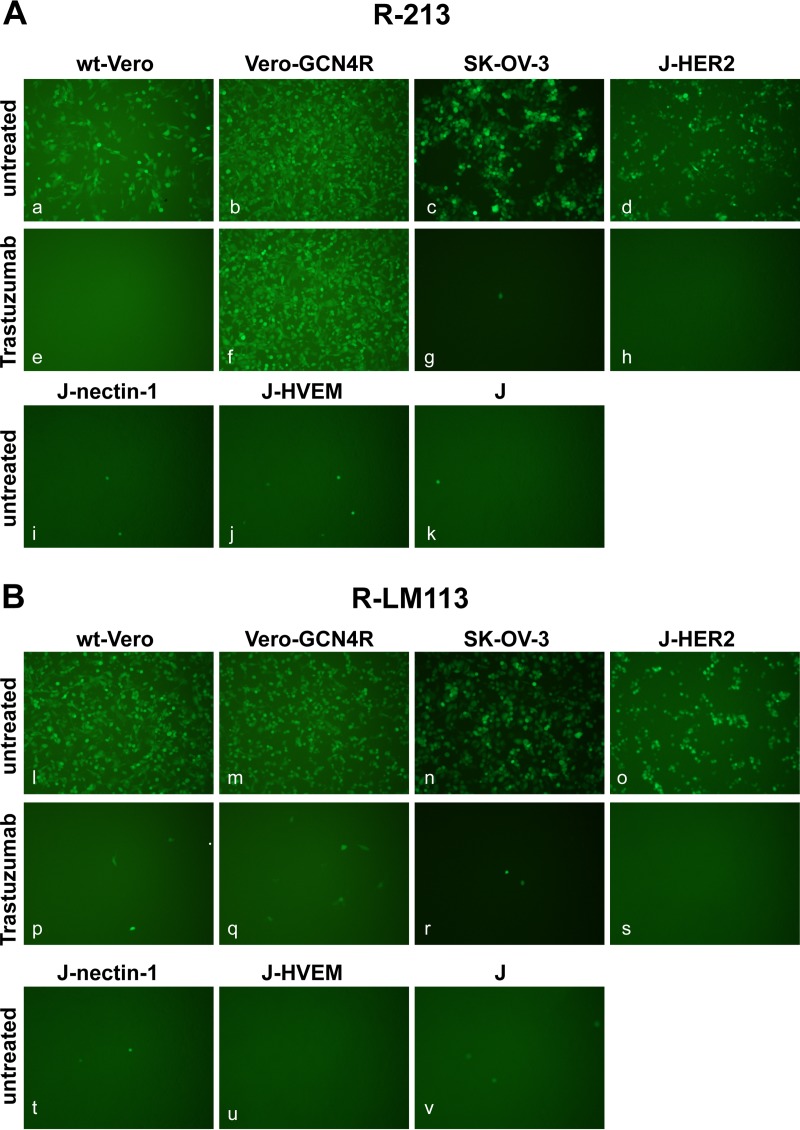
Double tropism of R-213 for Vero-GCN4R and HER2-positive cells. The indicated cells were infected with R-213 (A) or R-LM113 (B) at an MOI of 1 PFU/cell and monitored for EGFP expression by fluorescence microscopy 24 h postinfection. J-cells express no receptor for wt HSV; J-HER2, J-nectin-1, and J-HVEM express the indicated receptor. Cells in panels e to h and p to s were infected in the presence of the humanized anti-HER2 MAb trastuzumab at a concentration of 28 μg/ml. Some of the panels were adjusted as follows: 90% increase in brightness in panels a, b, c, e, f, g, l, m, n, p, q, and r.

To verify whether R-213 exhibits the expected dual tropism for GCN4R and HER2, we assayed its ability to infect the Vero-GCN4R cells, its wt-Vero counterpart, the HER2-positive SK-OV-3 cancer cells, and the J-HER2 cells. J-cells express no receptor for HSV and cannot be infected by wt HSV (27). J-nectin-1 and J-HVEM cells express the corresponding receptor (27) and were included as controls. The parental R-LM113 virus was included for comparison. R-LM113 carries the Δ6–38 deletion in gD, which detargets the virus tropism from the natural gD receptors HVEM and nectin-1. In place of the deleted sequences, R-LM113 carries the scFv to HER2. These same modifications in gD are present in both R-213 and R-LM113; hence, both viruses are retargeted to HER2 and detargeted from natural receptors (Table 1 summarizes their genotypic and tropism characteristics). The results shown in Fig. 2A demonstrate that R-213 infected Vero-GCN4R, SK-OV-3, and J-HER2 cells (Fig. 2A, panels b to d). The results in Fig. 2B, panels n and o, show that R-LM113 infected SK-OV-3 and J-HER2 cells, as expected. A striking result was the infection of wt-Vero cells by both viruses (Fig. 2A, panel a, and B, panel l). wt-Vero cells express the simian ortholog of HER2, and indeed infection of these cells with either R-213 and R-LM113 was inhibited by prior incubation of the cells with trastuzumab, the anti-HER2 human antibody from which the scFv to HER2 was derived (Fig. 2A, panel e, and B, panel p). Trastuzumab also inhibited infection of SK-OV-3 and J-HER2 cells with both R-213 and R-LM113, indicating that infection of these cells is through HER2. Infection of Vero-GCN4R cells with R-213 was only partly decreased by trastuzumab, providing evidence that this infection occurred in part independently of HER2 (Fig. 2A, panel f). As expected, R-213 failed to infect J-nectin-1, J-HVEM, and J-cells; thus, it maintained the detargeted phenotype exhibited by R-LM113 (Fig. 2A, panels i to k). We conclude that R-213 is retargeted to both the GCN4R and to HER2. Despite the fact that infection of Vero-GCN4R cells with R-213 was also highly efficient in the presence of trastuzumab, we cannot rule out that the infection of Vero-GCN4R cells was in part also enabled by HER2. The results indicate that it is possible to retarget HSV to two different target receptors, and the virus can use these receptors as portals of entry, independently of one another. The double retargeting was achieved by means of two different glycoproteins, gD and gH, as retargeting tools.

**TABLE 1:** Summary of genotypic modifications and tropism of recombinants employed in this study

Virus	HER2		GCN4		BAC sequence inserted in UL3-UL4 intergenic region^*a*^	Reference
	Viral gene in which scFv-HER2 is inserted	Retargeting to HER2	Viral gene in which GCN4 peptide is inserted	Retargeting to GCN4R		
R-213	gD_Δ6–38_	+	gH	+	+	This paper
R-LM113	gD_Δ6–38_	+	None	−	+	15
R-LM5	None	−	None	−	+	15

a EGFP gene was cloned within the BAC sequences.

### The Vero-GCN4R cells enable efficient replication and spread of GCN4-retargeted R-213.

**FIG 3:**
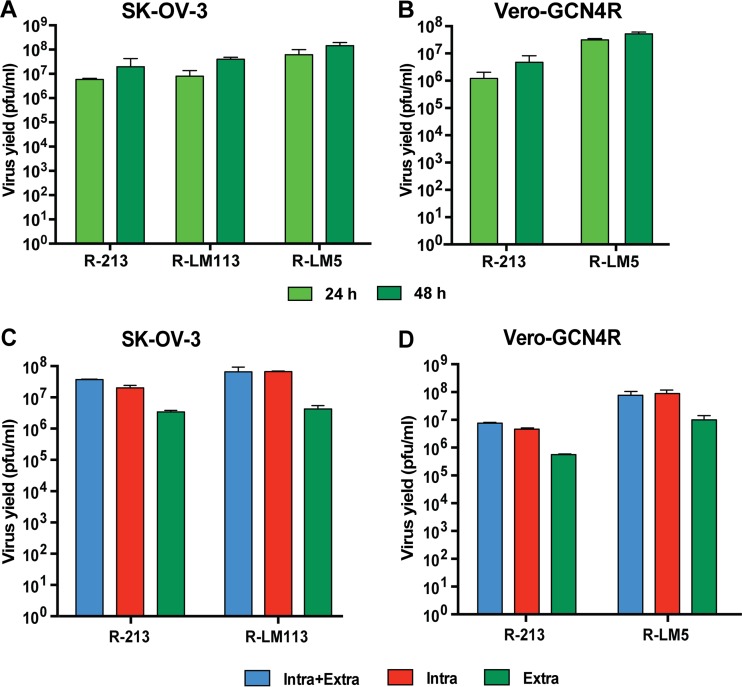
Yield of R-213, R-LM113, and R-LM5 in Vero-GCN4R cells or SK-OV-3 cells and release into the extracellular medium. (A and B) SK-OV-3 (A) and Vero-GCN4R (B) cells were infected with R-213. The extent of R-213 replication was compared to that of R-LM5 in Vero-GCN4R and to that of R-LM113 and R-LM5 in SK-OV-3 cells. Cells were infected at 0.1 PFU/cell, according to virus titers determined in Vero-GCN4R cells. Samples were harvested at 24 and 48 h after infection. Progeny virus was titrated in SK-OV-3 cells. Virus yields (in PFU per milliliter) are expressed as the means of three independent experiments ± SD. (C and D) SK-OV-3 (C) and Vero-GCN4R (D) cells were infected with R-213, R-LM113, and R-LM5 at 0.1 PFU/cell (inoculum titrated in SK-OV-3 cells). Samples were harvested at 48 h after infection. Progeny virus present in the extracellular media, in the cell-associated fractions, or in cell-associated plus medium samples was titrated in SK-OV-3 cells. Results are expressed as the means of three independent experiments ± SD.

A key feature for the employment of Vero-GCN4R cells for virus production is the extent of R-213 replication. Vero-GCN4R cells were infected with R-213 and also with R-LM5 for comparison. R-LM5 carries a wt gD (Table 1) and does not have any retargeting moiety. Similar to the retargeted viruses, it carries the BAC sequences and therefore is the wt counterpart of the retargeted viruses. The viral yields in SK-OV-3 cancer cells were also determined. Figure 3B shows that R-213 yield in Vero-GCN4R cells was about 1- to 1.5-logs lower than that of R-LM5 (Fig. 3B). In SK-OV-3 cells, the decrease in virus yield was exhibited also by R-LM113 (Fig. 3A). The data suggest that the gD modifications which retarget R-213 and R-LM113 to HER2 result in a somewhat-decreased viral replication. The difference in virus yield between R-213 and R-LM113 in SK-OV-3 cells was very low, implying that the insertion of the GCN4 peptide in gH caused a very minor effect on the replicative ability of R-213. We further quantified the extent of virus release into the extracellular medium. Cells were infected as described above for virus yield determinations. At 48 h after infection, cells and medium were harvested separately. Figure 3C shows that the extracellular virus/intracellular virus titer ratio was very similar for R-213 and for R-LM113 in SK-OV-3 cells. Figure 3D shows that the relative extracellular-to-intracellular virus titer ratios were very similar for R-213 and R-LM5 in Vero-GCN4R cells. Thus, Vero-GCN4R cells enable efficient virus replication and virus release into the medium.

**FIG 4:**
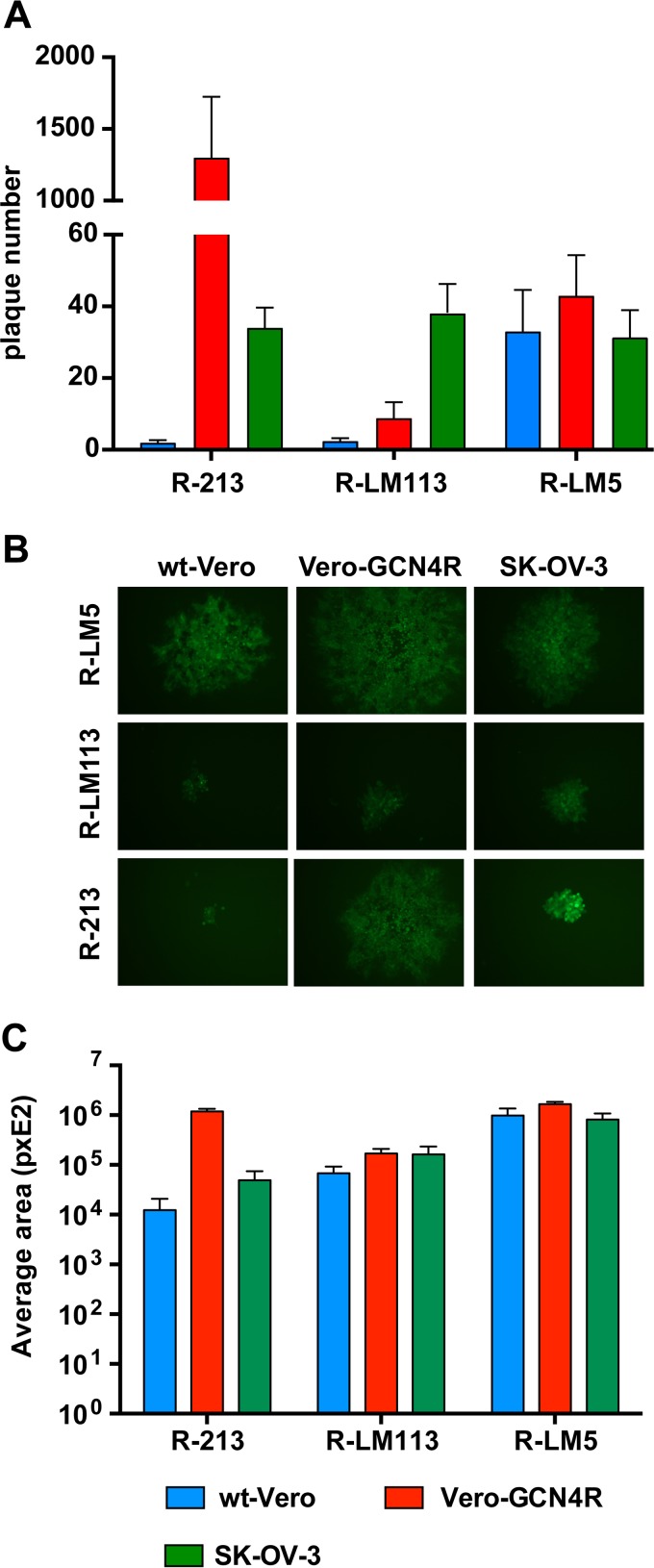
Plating efficiency of R-213 in different cell lines and relative plaque size. (A) Replicate aliquots of R-213, R-LM113, or R-LM5, containing the same amount of virus as titrated in SK-OV-3 cells, were plated in wt-Vero, Vero-GCN4R, and SK-OV-3 cells. The number of plaques was scored 3 days later. Results represent the means of five independent experiments ± SD. (B). Relative plaque size caused by R-213 in different cell lines. Representative plaques of R-213, R-LM113, and R-LM5 in wt-Vero, Vero-GCN4R, or SK-OV-3 cells at 3 days after infection. (C) Determination of plaque size. Plaque areas were measured by means of the Nis Elements-Imaging software (Nikon). Each result represents the average area of 5 plaques ± SD.

Next, we analyzed effects on cell-to-cell spread capability in Vero-GCN4R cells, as measured in a plaque assay. Replicate aliquots of each virus were plated on wt-Vero, Vero-GCN4R, and SK-OV-3 cells. R-213, but not R-LM113 or R-LM5, exhibited a much higher plating efficiency in Vero-GCN4R cells than in SK-OV-3 cells (Fig. 4A); the average data from 5 experiments showed a difference higher than 1 log (Fig. 4A). Representative plaque sizes are shown in Fig. 4B. The plaque size of R-213 in Vero-GCN4R cells was similar to, or even higher than, that in SK-OV-3 cells. Quantification of plaque areas is shown in Fig. 4C. Overall, R-213 exhibited a remarkably good ability to increase cell-to-cell spread in Vero-GCN4R cells, highlighting that the insertion of the GCN4 peptide in gH exerted no deleterious effect on the replication and spreading capacities of HER2-retargeted HSV.

### Cytotoxicity exerted by R-213.

**FIG 5:**
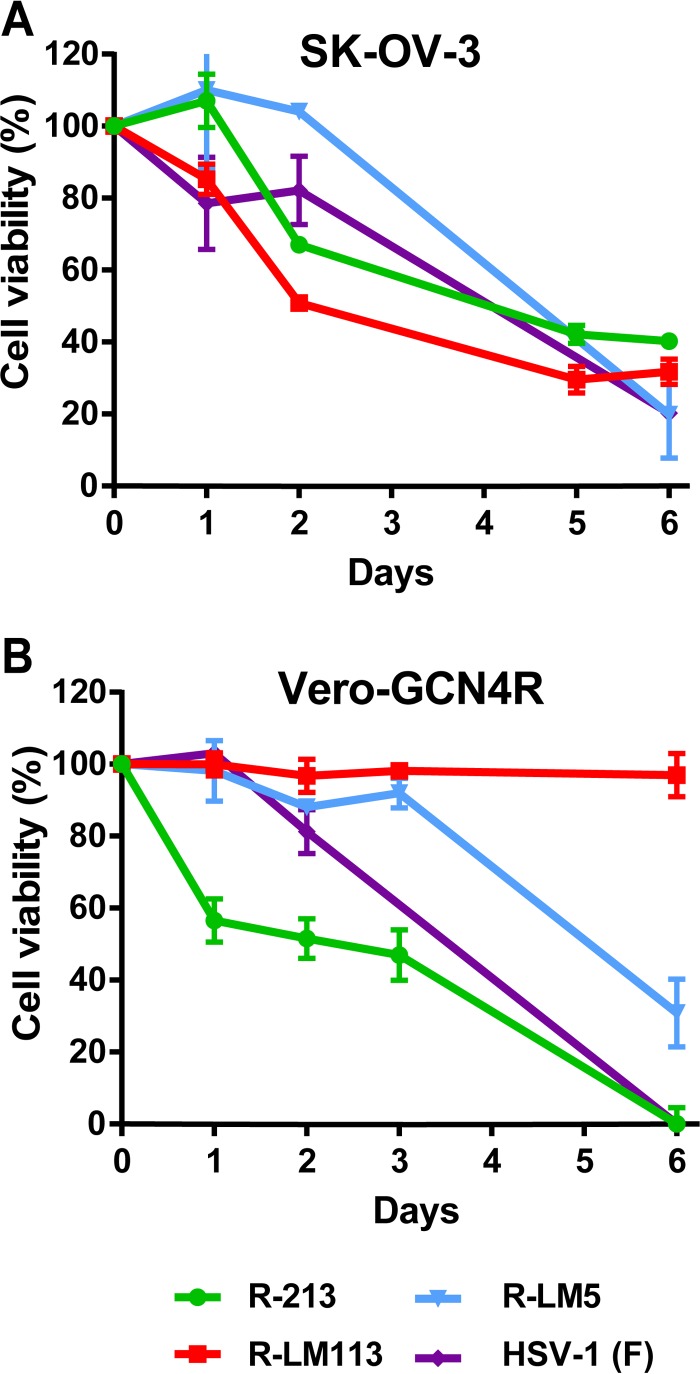
Cell-killing ability of R-213 in Vero-GCN4R and SK-OV-3 cells. (A) SK-OV-3 cells were infected with R-213 (3 PFU/cell), R-LM113 (3 PFU/cell), R-LM5 (1 PFU/cell), or HSV-1 (F) (1 PFU/cell). (B) Vero-GCN4R cells were infected with R-213 (6 PFU/cell), R-LM113 (6 PFU/cell), R-LM5 (6 PFU/cell), or HSV-1 (F) (3 PFU/cell). Cell viability was determined by using alamarBlue in triplicate samples. Each point represents the average result of triplicates ± the SD.

A key property for any candidate oncolytic HSV is its ability to kill cancer cells *in vitro*. Here, we compared the ability of R-213 to that of R-LM113 to kill SK-OV-3 cells, as measured in an alamarBlue reduction assay. Vero-GCN4R cells were included for comparison. SK-OV-3 or Vero-GCN4R cells were infected with R-213, R-LM113, R-LM5, and HSV-1 (strain F) at the indicated multiplicity of infection (MOI). alamarBlue was added at the indicated days after infection. The killing ability was determined as the percent reduction in the viability of the infected cell samples, relative to viability of uninfected cells. Figure 5 shows that the cytotoxic activities of R-213 and R-LM113 for the SK-OV-3 cells were very similar, and in general similar to that of the wt viruses. In contrast, R-213 but not R-LM113 was cytotoxic for Vero-GCN4R cells, in agreement with the infection and replication patterns described above. The killing ability of R-213 in Vero-GCN4R cells was higher than that of R-LM5 and HSV-1 (F). R-LM113 was not cytotoxic for Vero-GCN4R cells, in agreement with the limited infection in these cells. Thus, even in this assay, the insertion of the GCN4 peptide did not alter the cytotoxic ability of the HER2-retargeted HSV.

## DISCUSSION

In earlier work, we and others showed that it is possible to readdress HSV tropism by means of deletions in gD which remove critical residues for interaction with HVEM or nectin-1 and the replacement of deleted sequences with a ligand to a cancer receptor of choice (14–19). Recently, we also showed that gH can be a retargeting tool. The insertion in gH of the scFv to HER2, coupled with the deletion of aa 6 to 38 in gD, retargets HSV to HER2 (24). Here, we took advantage of the fact that HSV tropism can be modified via gD or via gH to address whether the same virus can be simultaneously readdressed to two different receptors, whether the virus can make use of two receptors independently of the other and, ultimately, to design a non-cancer cell line suitable for growth of clinical-grade retargeted HSV. We report that (i) it is possible to modify HSV tropism by inserting in gH the 20-aa short GCN4 peptide, (ii) the gH-mediated retargeting can be combined with the gD-mediated retargeting to HER2, as the double-retargeted HSV can use the GCN4 receptor and the HER2 receptor as alternative portals of entry to infect different sets of cells; (iii) the alternative usage of two receptors has enabled the development of a non-cancer cell line to grow retargeted oncolytic HSVs.

We successfully retargeted HSV tropism by insertion of a 20-aa-long peptide, made up of a 12-aa-long epitope plus bracketing residues and GS linkers. The scFv that forms the N terminus of the artificial GCN4R was derived from a monoclonal antibody that binds the epitope at very high affinity (20 pM) (25). The length of the GCN4 peptide is in sharp contrast with the length of the heterologous ligands employed earlier for gD retargeting, namely, interleukin-13, urokinase plaminogen activator, and scFvs to HER2 or to EGFR (17, 19, 28, 29). The latter varied in length from about 135 to 255 aa, and therefore carried defined structural domains. Our current results show for the first time that HSV retargeting does not require the insertion of structurally complex protein moieties and that it is sufficient to insert in gH a 20-aa peptide, possibly even a shorter one, to confer a novel tropism to HSV.

HSV can infect cells that express either GCN4R or HER2 as the sole receptor. The novel finding to emerge is that R-213 makes alternative use of the two receptors as portals of entry. Taking into account that the interaction with HER2 is via gD and the interaction with GCN4 receptor is via gH, the determinants of the viral tropism are alternatively gD or gH, depending on the specific receptor that the virus uses to infect each specific cell. This property opens the way to engineer oncolytic HSVs that are simultaneously retargeted to two different receptors of choice.

The capacity of a retargeted HSV to use alternative receptors of choice via gD or via gH has made it possible to grow the retargeted oncolytic HSV in non-cancer cells. A few properties of these cells are worth noting.

- A comparison of the extent of growth and cell-cell spread caused by R-213 versus R-LM113 in SK-OV-3 cells showed that R-213 was not hampered relative to R-LM113, indicating that the addition of the short GCN4 peptide in gH was not deleterious to gH functions. A comparison of R-213 growth and spread in Vero-GCN4R cells relative to that SK-OV-3 cells highlighted a somewhat lower titer in Vero-GCN4R cells, as expected, given that SK-OV-3 are cancer cells. Altogether, the Vero-GCN4R cell line exhibits promising properties for the *in vitro* growth of clinical-grade retargeted HSV in non-cancer cells.
- The GCN4 receptor is an artificial receptor that we generated by fusion of an scFv to the GCN4 peptide with domains II and III, TM, and the C tail of nectin-1. Nectin-1 does not play any role in GCN4R-mediated entry, for two reasons. First, it is well established that the only nectin-1 domain that mediates virus entry and interacts with gD is domain I (30–34); hence, the nectin-1 domains present in GCN4R do not play any role in HSV entry. Second, R-213 is detargeted from nectin-1 by virtue of the gD deletion, as shown by the lack of infection of J-nectin-1 cells.
- The strategy employed to design the Vero-GCN4R cell line differs from previous methods used to generate producer cell lines for retargeted adenovirus and measles virus (35, 36). The difference rests on the fact that, in previous examples, the same protein—adenovirus fiber or measles virus glycoprotein H—was modified for cancer retargeting and for targeting to the producer cell line. In contrast, here we made use of two different virion glycoproteins, gD for retargeting to cancer receptors and gH for retargeting to the producer cell line.
- The Vero-GCN4R cell line and gH retargeting via the GCN4 peptide can be applied to oncolytic HSVs retargeted not only to HER2 but also virtually to any cancer receptor of choice. Thus, the Vero-GCN4R cell line may be a universal cell line for cultivation of retargeted oncolytic HSVs.

The availability of a universal, non-cancer cell line for growth of clinical-grade retargeted oncolytic HSVs represents a step forward in the translational phase of these viruses.

## MATERIALS AND METHODS

### Generation of the Vero-GCN4R cell line.

To generate the chimeric receptor specific for the GCN4 peptide, a single-chain antibody specific for the peptide (Protein Data Bank accession number 1P4B) (25) was fused with domains II and III and domains of the transmembrane and C-tail of nectin-1. An HA epitope was added at the N terminus of the scFv for detection purposes. The plasmid carrying the artificial receptor was named scFv GCN4-nectin. The insert was synthesized *in vitro* and cloned in BamHI and XhoI restriction sites of a pcDNA3.1/Hygro(+) vector by using GeneArt (Thermo Fisher Scientific).

Vero cells (ATCC CCL-81) were transfected with scFv GCN4-nectin by means of Lipofectamine 2000 (Thermo Fisher Scientific) and selected for resistance to hygromycin B (400 μg/ml for 7 days). The selected cells were enriched with microbeads (Miltenyi Biotech) following incubation with an anti-HA antibody (Sigma-Aldrich Corporation) diluted 1:100 and with secondary anti-mouse antibodies (Jackson Laboratories). For the IFA, cells were reacted with an anti-HA antibody (Sigma-Aldrich Corporation) diluted 1:100 and secondary anti-mouse antibodies (Jackson Laboratories). Single-cell clones were obtained by limiting dilution. Clones were checked for stability for up to 40 passages in cell culture by flow cytometry (BD Accuri).

### Viruses.

The following recombinant viruses were previously described (Table 1 summarizes patterns of genotypes and tropism). R-LM5 carries the wt gD open reading frame (ORF), the BAC sequences cloned in the UL3-UL4 intergenic region, as in the parental pYeBac102 (37), and the enhanced green fluorescent protein (EGFP) ORF under the control of the α27 promoter cloned within the BAC sequences (15). Since it is not detargeted/retargeted, it is the wt counterpart of R-LM113 and R-213. R-LM113 carries a HER2-retargeted gD and is otherwise identical to R-LM5. The retargeted gD was engineered by deletion of aa 6 to 38 of mature gD and replacement with the scFv to HER2 derived from trastuzumab (38). The deletion of aa 6 to 38 removes critical residues for interaction with HVEM and nectin-1; hence, it detargets the virus tropism from the natural receptors.

### Engineering of R-213.

R-213 was derived from R-LM113 by insertion of the GCN4 peptide in gH, between aa 23 and 24; hence, it carries the HER2-retargeted gD. The amino acid sequence of the GCN4 peptide was GSKNYHLENEVARLKKLVGS (Fig. 1B). The core YHLENEVARLKK residues represent the epitope recognized by the single-chain antibody C11L34-H6 (PDB ID 1P4B) (25). In R-213, the GCN4 peptide was preceded and followed by GS linkers (Fig. 1B).

The starting material for the engineering of R-213 was BAC LM113, (15). The engineering was performed in bacteria by means of *galK* recombineering, in two steps (24, 39). In the first step, the *galK* cassette, with homology arms to gH, was inserted in the selected site of gH (between aa 23 and 24). In the second step, the *galK* insert was replaced with the GCN4 peptide. The resulting recombinant BAC DNA was then transfected in Vero-GCN4R cells, for virus reconstitution. In detail, to carry out the first step, the *galK* cassette, with homology arms to gH, was amplified by means of primers gH6_galK_f, ATGCGGTCCATGCCCAGGCCATCCAAAAACCATGGGTCTGTCTGCTCAGTCCTGTTGACAATTAATCATCGGCA, and gH5_galK_r, TCGTGGGGGTTATTATTTTGGGCGTTGCGTGGGGTCAGGTCCACGACTGGTCAGCACTGTCCTGCTCCTT, using plasmid pgalK as the template. The PCR-amplified *galK* cassette was then electroporated into SW102 bacteria, which carry the BAC LM113, to generate BAC 212. To exclude *galK* false-positive colonies, the recombinant clones were plated on MacConkey agar base plates supplemented with 1% galactose and 12 μg/ml chloramphenicol and checked by colony PCR with primer galK_129_f, ACAATCTCTGTTTGCCAACGCATTTGG, and galK_417_r, CATTGCCGCTGATCACCATGTCCACGC. To carry out the second step, a cassette encoding the GCN4 peptide (GenBank accession number AF416613.1), bracketed by the downstream and upstream Gly-Ser linkers and by homology arms to gH, was generated through annealing and extension of the partially overlapping oligonucleotides GCN4gH_23_42_fB, TCGTGGGGGTTATTATTTTGGGCGTTGCGTGGGGTCAGGTCCACGACTGGGGATCCAAGAACTACCACCTGGAGAACGAGGTGGCCAGACTGAAGAAGCTGGTGGGCAGC, and GCN4gH_23_24_rB, ATGCGGTCCATGCCCAGGCCATCCAAAAACCATGGGTCTGTCTGCTCAGTGCTGCCCACCAGCTTCTTCAGTCTGGCCACCTCGTTCTCCAGGTGGTAGTTCTTGGATCC. The oligonucleotides contained a silent BamHI restriction site, for screening purposes. The amplimer encoding the GCN4 cassette, with homology arms to gH, was electroporated into SW102 bacteria carrying BAC 212. The recombinant BAC was named BAC 213. Positive bacterial clones were checked by means of BamHI restriction analysis on colony PCR fragments amplified with primers gH_ext_r, GTTTCTTCCTTTTCCCCACCCCACCCC, and gH_2176_2200_f, CAGGTAGGTCTTCGGGATGTAAAGC. BAC 213 DNA (500 ng) was then transfected into Vero-GCN4R cells by use of Lipofectamine 2000 (Life Technologies) in order to reconstitute the R-213 recombinant virus. Virus growth was monitored by fluorescence of the EGFP marker, cloned within the BAC sequences. The recombinant gH of R-213 was checked by sequencing. Viral stocks were generated and titrated in Vero-GCN4R and SK-OV-3 cells.

### Tropism of R-213.

The indicated cells were infected with R-213 or R-LM113, at 1 PFU/cell, and monitored 24 h later with a Nikon Eclipse TS100 fluorescence microscope. Infection was carried out in the presence of monoclonal antibody (MAb) to HER2 (trastuzumab) at a concentration of 28 μg/ml, where indicated.

### Determination of virus growth and extent of viral progeny release.

Vero-GCN4R and SK-OV-3 cells were infected with R-LM5, R-LM113, or R-213 at 0.1 PFU/cell. Unabsorbed virus was inactivated by rinsing the cells with a pH 3 solution (40 mM citric acid, 10 mM KCl, 135 mM NaCl). Replicate cultures were frozen at 24 and 48 h after infection. Progeny virus (intracellular plus extracellular) was titrated in SK-OV-3 cells. Results are expressed as the mean findings of three independent experiments ± the standard deviation (SD).

In virus release experiments, replicate cultures of Vero-GCN4R or SK-OV-3 cells infected with R-LM5, R-LM113, or R-213 at 0.1 PFU/ml were harvested 48 h after infection as cell lysates plus medium. Alternatively, medium and cell-associated fractions were harvested separately. Progeny virus was titrated in SK-OV-3 cells. Results are expressed as the mean findings of three independent experiments ± SD.

### Plating efficiency and relative plaque size.

Replicate aliquots of R-LM5, R-LM113, or R-213 were plated on wt-Vero, Vero-GCN4R, and SK-OV-3 cells, and the number of plaques was counted 3 days later. Results represent the mean findings of three independent infections ± SD. For plaque size determination, pictures of 5 individual plaques from each of the above samples were taken 3 days after infection. Plaque areas were measured with Nis Elements-Imaging software (Nikon). Each result represents mean areas ± SD.

### Cytotoxicity assay.

SK-OV-3 and Vero-GCN4R cells were seeded in 96-well plates (8 × 10^3^ cell/well) and infected with R-LM5, R-LM113, or R-213 (6 or 3 PFU/cell) or mock infected. alamarBlue (10 μl/well; Life Technologies) was added to the culture medium at the indicated times after infection and cultures were incubated for 4 h at 37°C. Plates were read at 560 and 600 nm with GloMax Discover system (Promega Corporation). For each time point, cell viability was expressed as the percentage of alamarBlue reduction in infected versus uninfected cells, after subtraction of the background value (medium alone). Each point represents the average results of at least triplicate samples ± SD.
